# Nonbacterial thrombotic endocarditis associated with cancer of unknown origin complicated with thrombus in the left auricular appendage: case report

**DOI:** 10.1186/1476-7120-9-8

**Published:** 2011-02-28

**Authors:** Kazuko Norisada, Hidekazu Tanaka, Tetsuari Onishi, Akihiro Kaneko, Takayuki Tsuji, Kohei Yamawaki, Keiko Ryo, Kazuhiro Tatsumi, Kensuke Matsumoto, Natsuko Miura, Jun Saegusa, Yukiko Morinaga, Shigeo Hara, Hiroya Kawai, Ken-ichi Hirata

**Affiliations:** 1 Division of Cardiovascular Medicine, Department of Internal Medicine, Kobe University Graduate School of Medicine, Kobe, Japan, 7-5-2, Kusunoki-cho, Chuo-ku, Kobe, 650-0017, Japan; 2 Department of Clinical Pathology and Immunology, Kobe University Graduate School of Medicine, Kobe, Japan, 7-5-2, Kusunoki-cho, Chuo-ku, Kobe, 650-0017, Japan; 3 Department of Pathology,Kobe University Graduate School of Medicine, Kobe, Japan 7-5-2, Kusunoki-cho, Chuo-ku, Kobe, 650-0017, Japan

## Abstract

A 63-year-old man was admitted to our hospital with a complaint of right lateroabdominal pain. He was diagnosed with metastatic colon cancer, and then developed multiple brain embolic infarctions 7 days after admission. Transesophageal echocardiography showed that mobile, echo-dense masses were attached to the anterior and posterior mitral valve leaflet. Furthermore, there was a thrombus in the left auricular appendage despite sinus rhythm. These findings led to a diagnosis of suspected infectious endocarditis with subsequent multiple brain infarctions. The patient's general condition worsened and he died 13 days after admission. An autopsy was performed, and, while poorly differentiated cancer was observed in multiple organs, no primary tumor could be identified. Histological analysis showed that the masses of the mitral valve consisted mainly of fibrin without bacteria or oncocytes. This patient was therefore diagnosed with nonbacterial thrombotic endocarditis associated with cancer of unknown origin complicated with thrombus in the left auricular appendage.

## Background

Nonbacterial thrombotic endocarditis (NBTE) is a rare condition associated with cancer and other illnesses with hypercoagulable states, including septicemia and autoimmune disease [1]. It causes aseptic masses of fibrin and platelets usually along the line of previously undamaged heart valves. The vegetations in NBTE are friable and tend to detach and cause extensive infarction more readily than the vegetations observed in infective endocarditis. Nearly half of patients present clinically with systemic emboli, with cerebral emboli occurring most commonly [2]. A diagnosis of NBTE is most commonly made at autopsy and is rarely made during life. This report concerns a case of NBTE associated with cancer complicated with thrombus in the left auricular appendage and multiple cerebral infarctions.

## Case presentation

**Figure 1:**
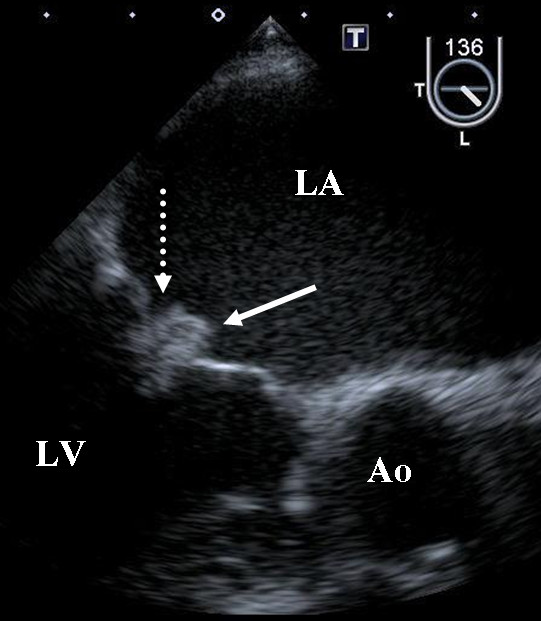
**Masses of the mitral valve**. **Ao, aorta; LA, left atrium; LV, left ventricle**. Transesophageal echocardiography showed mobile, echo-dense masses measuring 3 × 7 mm and 6 × 4 mm attached, respectively, to the anterior (white arrow) and posterior (dotted white arrow) mitral valve leaflet.

**Figure 2:**
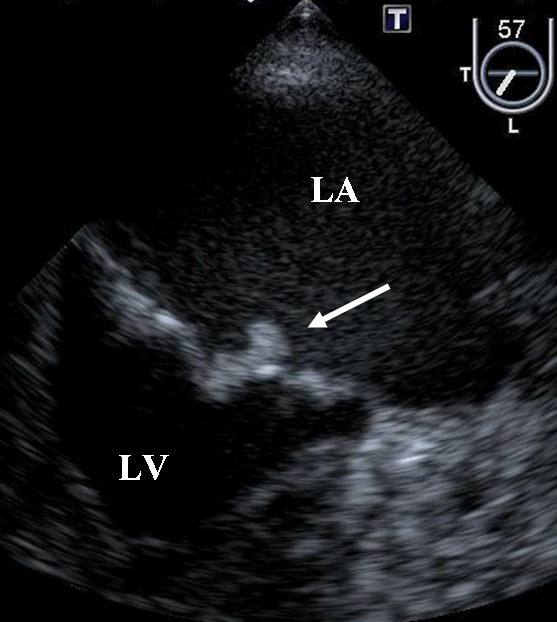
**Masses of the mitral valve**. All abbreviations and descriptions as in Figure 1.

**Figure 3:**
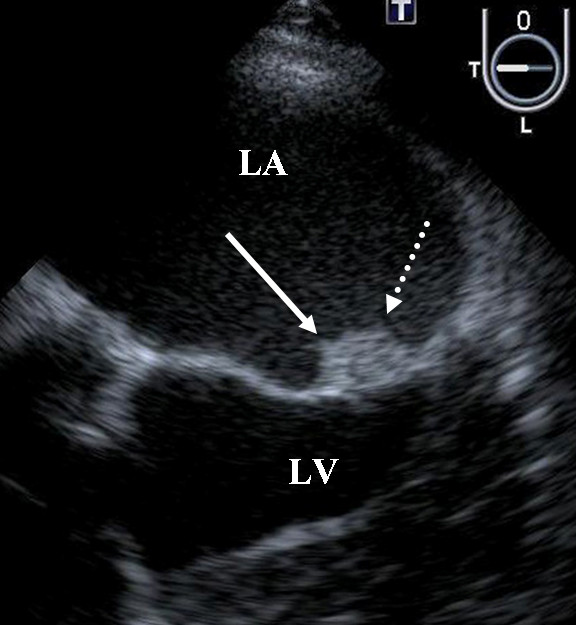
**Masses of the mitral valve**. All abbreviations and descriptions as in Figure 1.

**Figure 4:**
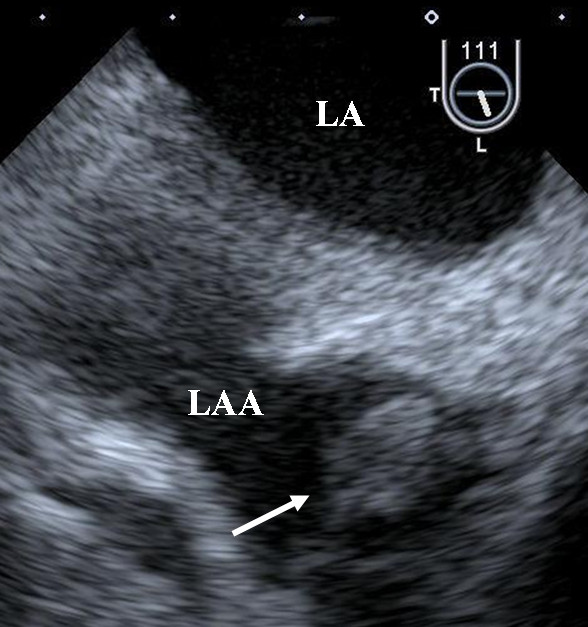
**A thrombus in the left autricular appendage**. **LA, left atrium; LAA, left auricular appendage**. A thrombus (12 × 8 mm) was observed in the left auricular appendage (white arrow).

A 63-year-old man was admitted to our hospital with a complaint of right lateroabdominal pain. He had a history including hypertension, hyperlipidemia and chronic rheumatoid arthritis. Physical examination found his blood pressure was 130/74 mmHg and pulse was regular at 72 beats/min. No heart murmur was heard and the lungs were clear on auscultation. Chest X-ray and 12-lead electrocardiography findings were normal. Laboratory test results showed D-dimer of 22.8 μg/ml and serum carcinoembryonic antigen of 1073 ng/ml. Colon contrast enema and colonoscopy revealed the stenosis of the rectum and transverse colon. Endoscopic ultrasonography disclosed that a tumor in the muscular layer of the rectum had invaded from the peritoneal cavity, but that the mucosa and submucous layer were intact. These findings led to a diagnosed of metastatic colon cancer. Seven days after admission, aphasia occurred, and magnetic resonance imaging of the brain confirmed the presence of multiple embolic infarctions. Transesophageal echocardiography to evaluate the neurological symptoms indicated that two mobile, echo-dense masses measuring 3 × 7 mm and 6 × 4 mm, were attached to the anterior and posterior mitral valve leaflet, respectively (Figure 1, 2 and 3, Additional file 1; Video 1). However, no significant mitral stenosis or regurgitation was observed (Additional file 2; Video 2). Furthermore, a thrombus (12 × 8 mm) was detected in the left auricular appendage (Figure 4, Additional file 3; Video 3). Other sites of thomboembolic dissemination were not documented. The patient was therefore suspected of having infectious endocarditis with subsequent multiple brain infarctions. However, we also considered the possibility of aseptic endocarditis because serial blood cultures were sterile and there were no clinical signs of infection. Anticoagulation therapy with heparin was administrated, but the patient's general condition worsened and he died 13 days after admission.

**Figure 5:**
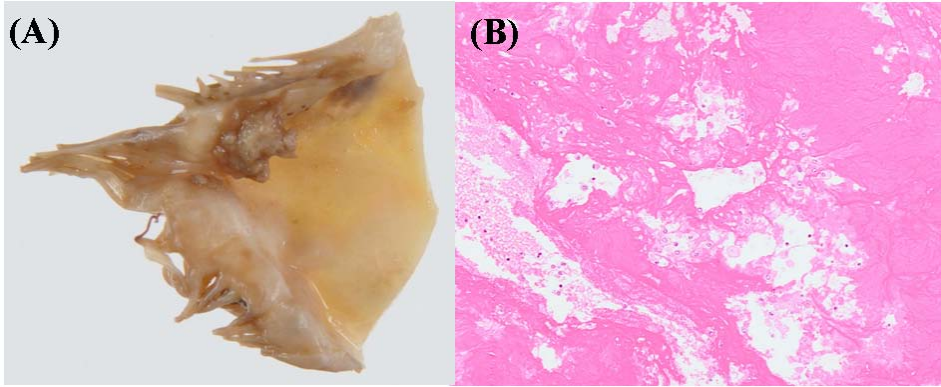
**Pathological findings**. **(A) **Gross examination of the vegetation removed from the mitral valve leaflet. **(B) **Histological examination showed the vegetation is composed mainly of fibrin without bacteria or oncocytes (hematoxylin-eosin staining × 100).

The autopsy showed poorly differentiated cancer in multiple organs, including the serous side of the transverse colon, the sigmoid colon, rectum, pancreas head, bladder, mediastinum, peritoneum and epicardium. However, no primary tumor could be identified. The masses of the mitral valve were histologically composed mainly of fibrin without bacteria or oncocytes (Figure 5). This patient was therefore ultimately diagnosed with NBTE associated with cancer of unknown origin.

## Discussion

The case presented here demonstrates an uncommon paraneoplastic disease that a patient with NBTE associated with cancer of unknown origin can be complicated with thrombus in the left auricular appendage and multiple cerebral infarctions.

NBTE, characterized by the presence of platelet-fibrin deposits on cardiac valves is known to be associated with hypercoagulable states, particularly in patients with malignant tumors [1]. The aseptic masses on cardiac valves of NBTE are friable and tend to detach and cause extensive infarction more readily than the vegetations observed in infective endocarditis. Nearly half of all NBTE patients present clinically with systemic emboli, most commonly cerebral emboli [2]. All complications of NBTE lead to severe damage to multiple organs and result in a poor outcome. The fact that a diagnosis of NBTE is usually made at autopsy indicates that making a definitive antemorterm diagnosis is very difficult. Although the antemorterm diagnosis is typically made by means of echocardiography, it can be difficult to distinguish between infective endocarditis and NBTE on the basis of echocardiographic features alone. However, such a distinction is of paramount importance in terms of selecting of the most appropriate therapeutic strategy. Various characteristics of NBTE have been reported before. The most commonly affected valves are the aortic valve, the mitral valve, and a combination of the two valves [3]. A large-scale study reported a mitral valve predominance of 64%, followed by aortic valve (24%) and both valves (9%) [4]. Platelet-fibrin deposits are commonly located on the atrial surface of the mitral and tricuspid valves as well as on the ventricular surface of the aortic and pulmonic valves. Valvular vegetations along coaptation lines without leaflet destruction (regurgitation), simultaneous occurrences of venous thromboembolism, negative blood cultures, and absence of clinical signs of infection are all suggestive of NBTE [5]. In the present case, thrombus in the left auricular appendage was complicated. A left atrial thrombus is usually associated with atrial fibrillation or mitral stenosis but is very infrequently detected in the presence of sinus rhythm [6,7]. In addition to above-mentioned characteristics of NBTE, the simultaneous presence of intracardiac thrombus may also be suggestive of NBTE.

NBTE has been reported in 4% of patients with all types of end-stage cancer, and the aseptic masses on cardiac valves of NBTE are very friable and therefore embolize easily. NBTE must be taken into account for patients with a background of cancer, with cerebral embolism due to unknown causes, and with negative blood cultures and absence of clinical signs of infection [8].

## Conclusions

In summary, NBTE is associated with hypercoagulable states, particularly in patients with malignant tumors, and it causes systemic embolism readily. NBTE must be taken into account for patients with a background of cancer, with cerebral embolism due to unknown causes, and with negative blood cultures and absence of clinical signs of infection.

## Abbreviations

NBTE: nonbacterial thrombotic endocarditis;

## Consent

Written informed consent was obtained from the kin of the patient for publication of this case report and any accompanying images. A copy of the written consent is available for review by the Editor-in-Chief of this journal.

## Competing interests

The authors declare that they have no competing interests.

## Authors' contributions

KN designed the study, carried out subject recruitment, performed echocardiography, analysed the data, and wrote the manuscript. HT, TO, AK, TT, KY, KR KT, KM, NM, JS, YM, SH, HK and KH assisted recruitment and manuscript revision.

All authors read and approved the final manuscript.

## Supplementary Material

Additional file 1 **Video 1. Transesophageal echocardiographic view of the mitral valve**. Transesophageal echocardiography showed mobile, echo-dense masses measuring 3 × 7 mm and 6 × 4 mm attached, respectively, to the anterior and posterior mitral valve leaflet. Click here for file

Additional file 2 **Video 2. Transesophageal color Doppler echocardiographic image of the mitral valve**. Transesophageal echocardiography showed that mitral regurgitation was trivial. Click here for file

Additional file 3 **Video 3. Transesophageal echocardiographic view of the left auricular appendage**. A thrombus (12 × 8 mm) was observed in the left auricular appendage. Click here for file
